# Beat-to-beat blood pressure measurement using a cuffless device does not accurately reflect invasive blood pressure

**DOI:** 10.1016/j.ijchy.2020.100030

**Published:** 2020-04-14

**Authors:** Mohammed A Moharram, Luke C Wilson, Michael JA Williams, Sean Coffey

**Affiliations:** Department of Medicine - HeartOtago, Dunedin School of Medicine, University of Otago, Dunedin, New Zealand

**Keywords:** Cuffless blood pressure, Pulse arrival time, Continuous blood pressure monitor, SOMNOtouch NIBP, BP, blood pressure, BtB, beat to beat, cBP, brachial cuff blood pressure, DBP, diastolic blood pressure, iBP, invasive blood pressure, PTT, pulse transit time, SBP, systolic blood pressure

## Abstract

**Background:**

The availability of an accurate continuous cuffless blood pressure (BP) monitor would provide an alternative to both invasive continuous BP and 24-h intermittent cuff-based BP monitors. We investigated the accuracy of a cuffless beat to beat (BtB) device compared to both invasive BP (iBP) and brachial cuff BP (cBP) measurements.

**Methods:**

Patients undergoing clinically indicated coronary angiography (CA) and/or percutaneous coronary intervention (PCI) were recruited. After calibration to an initial cBP reading, BP was measured simultaneously using a BtB device (SOMNOtouch NIBP), brachial artery iBP, and cBP at two time points.

**Results:**

The study was terminated early due to a significant bias. Recordings from 14 participants (11 males, mean age 68.4 years) were analysed. Readings from BtB BP were higher than iBP. The bias between BtB BP and iBP was 34.3 mmHg (95%CI: 27.0, 41.5) and 23.6 mmHg (95%CI: 16.8, 30.4) for SBP and DBP respectively. A similar bias was seen between BtB BP and cBP, but cBP and iBP were largely in agreement.

**Conclusions:**

In patients undergoing CA/PCI, significant differences were detected between BtB BP and both invasively measured and cuff BP. The non-invasive BtB BP measurement device tested is not suitable for clinical or research use.

## 1 Introduction

Raised blood pressure (BP) is one of the major causes of health loss worldwide [1]. Short-term and long-term BP variability, in addition to absolute BP values, could be related to adverse events in patients with hypertension [2,3]. Currently, most available non-invasive BP measurement devices are cuff based and rely on auscultatory or oscillimetric methods [4]. Non-invasive 24-h BP monitoring cuff based devices allow intermittent BP measurement; however, these devices are relatively bulky and are reliant on using a cuff [5,6]. In addition, clinical scenarios with rapidly changing BP currently rely either on frequent cuff based BP measurement, which may not have the required temporal resolution, or invasive BP measurement. Therefore, there is a need for an accurate, reliable and affordable non-invasive cuffless continuous BP monitoring technology [7].

The concepts and methods of cuffless BP measurement have been discussed for decades [8]. However, the development of cuffless BP measurement technology has been increasingly studied over recent years [[9], [10], [11]]. One of the concepts underpinning cuffless BtB continuous BP monitoring is pulse transit time (PTT) [7]. The PTT measures the time between the R wave detected on the electrocardiogram (ECG) and the pulse wave mechanical activity at a peripheral site on the arterial tree [6,7]. In this study, we aimed to investigate the accuracy of a device measuring continuous BtB BP using PTT: the SOMNOtouch NIBP system (Somnomedics GmbH, Randersacker, Germany) [12] in comparison to invasive BP (iBP) and oscillimetric brachial cuff BP (cBP) measurements.

## 2 Materials and methods

### 2.1 Subjects

This was a prospective study including patients >18 years old undergoing clinically indicated coronary angiography (CA) and/or percutaneous coronary intervention (PCI). Patients with atrial fibrillation (AF) or vascular lesions affecting the upper limb arterial tree were excluded from this study. Informed consent was obtained from all participants. The study protocol conforms to the ethical guidelines of the 1975 Declaration of Helsinki and was approved by the University of Otago Health Ethics Committee (reference: H19/024).

### 2.2 Device (SOMNOtouch NIBP)

The device consists of three ECG leads and a peripherally placed photoplethysmogram (PPG), both of which are connected to a watch-like unit that is placed at wrist level. The device was placed around the patient's left wrist during CA/PCI. PPG is an optic-based technique used in pulse oximetry to measure arterial oxygen saturation (SpO2) [13,14]. This technique is based on using a light-emitting diode (LED) and a photodetector to measure either the amount of light transmitted through (transmission-mode PPG) or reflected from the tissue (reflectance-mode PPG); this measured amount of light is then used to estimate oxygen saturation. Additionally, the measured reflected or transmitted amount of light is related to the instantaneous change in blood volume and can be used for PTT estimation [13,14].

The PPG sensor was placed around the index finger. The device manufacturer provided different sizes of the PPG sensor; appropriate sensor size ensures proper contact of the sensor with the patient's finger without having high contact pressure. The ECG was connected according to the manufacturer's instructions. The device estimates BP based on beat to beat measurement of PTT. PTT is calculated as the time interval between the R wave on the ECG detected through the connected ECG leads and the pulse wave detected by the PPG on the patient's finger. SBP and DBP are estimated using a nonlinear model [15]. BP was measured using the brachial cuff before the procedure to calibrate the SOMNOtouch NIBP according to the manufacturer instructions.

### 2.3 Brachial cuff oscillimetric BP measurement

The brachial cuff was placed around the left arm. BP was measured using the oscillimetric cBP method integrated into the cardiac catheterisation system (Artis zee with PURE®, Siemens AG, Munich, Germany).

### 2.4 Invasive BP measurements

BP was measured using an invasive BP line; pressure transducer was placed in a position that was at the approximate level with the heart and was set to zero by obtaining atmospheric pressure. The line was connected to a 5 French catheter inserted via the right radial artery with the tip placed at the level of the brachial artery.

### 2.5 Validation protocol

Two measurement points were taken; with the time stamp defined, measurements of both cBP and iBP were simultaneously recorded while corresponding BtB BP reading defined by the time stamp was later extracted and recorded. The first measurement point was taken after radial artery access while second measurement point was taken after the completion of the procedure.

### 2.6 Statistical analysis

For each measurement modality, two variables were analysed: SBP and DBP. Both SBP and DBP variables contained BP measurements at the two time points (before and after the CA/PCI procedure). To assess agreement between iBP and BtB BP measurements, we used Bland-Altman analysis. A univariate linear regression model was fitted to assess proportional bias between measurements. For the comparison of the differences in the mean between measurement modalities, paired two-tailed Student's t-test was used. Pearson correlation was used to assess correlations between variables. Statistical analyses were conducted using R (R version 3.5.3, RStudio Version 1.1.383, RStudio, Inc., Boston, MA, USA). Alpha of 0.05 was used as the cut-off for significance. Based on an expected mean of differences of 10 mmHg with a standard deviation of the mean of 5 mmHg, a sample size of 50 participants was estimated to provide at least 80% power to define difference at this level of significance.

## 3 Results

**Fig. 1:**
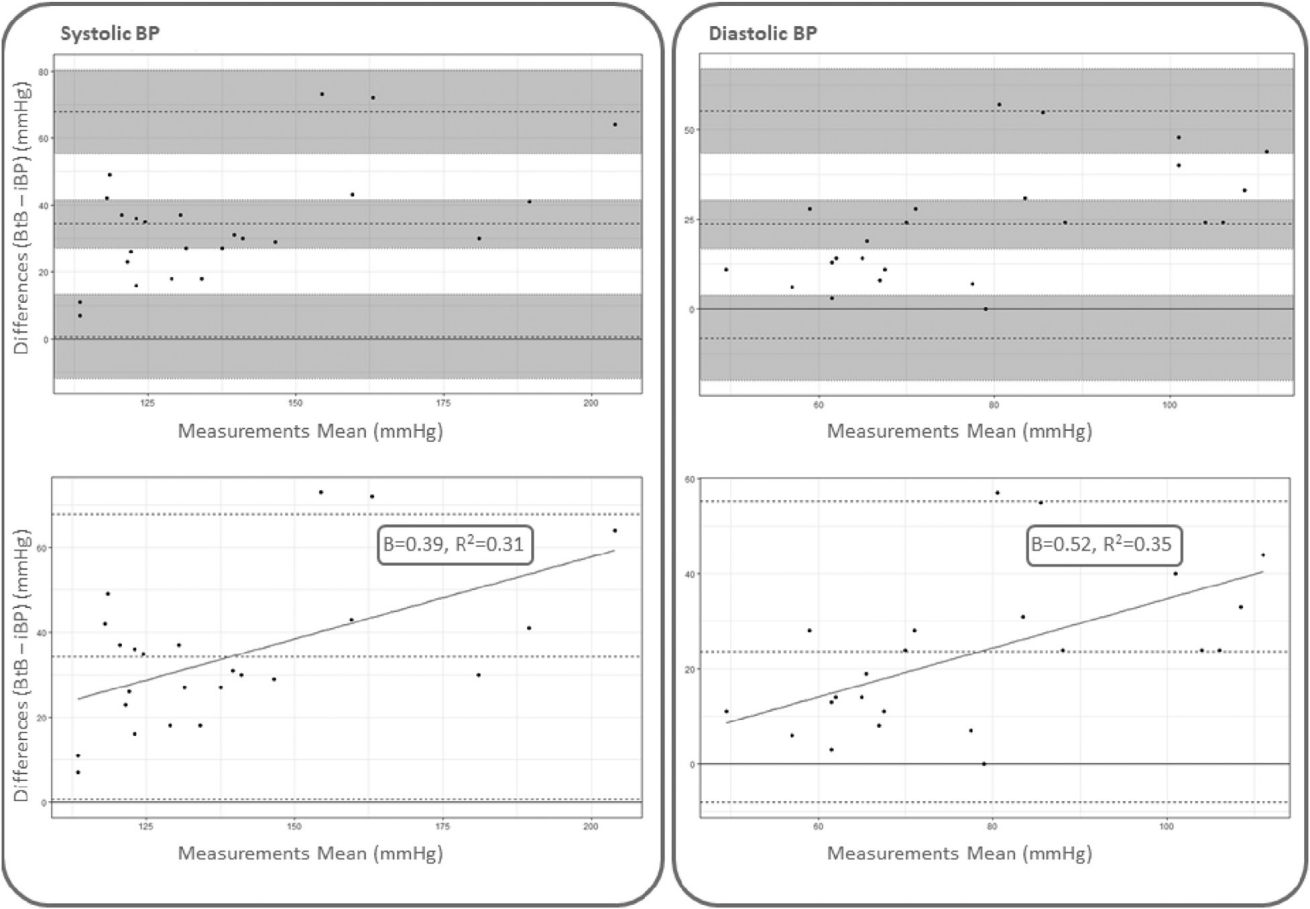
**Bland-Altman plot for comparison of beat to beat BP and invasive BP.** Bland-Altman plots comparing beat to beat BP to invasive BP. The upper plots show in grey the bias, upper limit of agreement, and lower limit of agreement with corresponding 95% confidence intervals for both SBP (left) and DBP (right). The lower plots show Bland-Altman plots comparing beat to beat BP to invasive BP with fitted univariate regression line between measurements differences and measurements means (mmHg). BP, blood pressure; BtB, beat to beat BP; iBP, invasive BP.

The study was terminated early after enrolment of 19 patients, due to larger than expected differences being identified. BtB BP measurements were satisfactorily acquired for 14 patients; five BtB recordings were excluded due to unsatisfactory quality or technical failure. Characteristics of the study participants are presented in Table 1. The bias between BtB BP and iBP was 34.3 mmHg (95%CI: 27.0, 41.5) and 23.6 mmHg (95%CI: 16.8, 30.4) for SBP and DBP respectively (Fig. 1). There was a proportional bias between BtB BP and iBP measurements with wider differences detected at higher readings (Fig. 1). The correlation coefficients between iBP and BtB BP were 0.84 and 0.71 for SBP and DBP respectively.

**Table 1:** Characteristics of the study population (n = 14).

**Age** (years, mean(SD))		68.4 (10.0)
**Sex** (male (n(%)))		11 (79%)
**HTN** (n(%))		12 (86%)
**Diabetes** (n(%))		2 (14%)
**Current smoker (n(%))**		0 (0%)
**Indication for CA/PCI**		
IHD (n(%))		10 (71%)
Others (n(%))		4 (29%)
**Invasive Blood Pressure (mmHg)**		
First measurement	SBP	122.7 (20.4)
	DBP	67.8 (16.3)
Second Measurement	SBP	128.2 (23.1)
	DBP	69.4 (14.7)
**Brachial Cuff Blood Pressure (mmHg)**		
First Measurement	SBP	119.8 (24.7)
	DBP	74.3 (12.8)
Second Measurement	SBP	130.8 (26.4)
	DBP	76.4 (13.2)
**Beat to Beat Blood Pressure (mmHg)**		
First Measurement	SBP	158.1 (29.3)
	DBP	93.9 (23.1)
Second Measurement	SBP	161.4 (36.0)
	DBP	96.2 (24.3)
**Medications**		
ACEi/ARBs (n(%))		10 (71%)
Beta blockers (n(%))		7 (50%)
Calcium Channel Blockers (n(%))		3 (21%)
Antiplatelets (n(%))		10 (71%)
Anticoagulants (n(%))		1 (7%)
Diuretics (n(%))		3 (21%)
Statins (n(%))		8 (57%)

HTN, hypertension; CA, coronary angiography; PCI, percutaneous coronary intervention; IHD, ischemic heart disease; SBP, systolic blood pressure; DBP, diastolic blood pressure; ACEi, angiotensin converting enzyme inhibitors; ARBs, angiotensin receptor blockers.

The bias between BtB SBP and cBP SBP was 30.6 mmHg (95%CI: 22.4, 38.8) with upper limit of agreement 70.2 mmHg (95%CI: 56.1, 84.4) and lower limit of agreement −9.1 mmHg (95%CI: −23.2, 5.1); The bias between BtB DBP and cBP DBP was 15.2 mmHg (95%CI: 9.0, 21.5) with upper limit of agreement 45.7 mmHg (95%CI: 34.8, 56.6) and lower limit of agreement −15.2 (95%CI: −26.1, −4.4). The Pearson correlation coefficients between BtB BP and cBP were 0.76 and 0.82 for SBP and DBP respectively. There was minimal bias between cBP and iBP; the bias was 0.1 mmHg (95%CI: −4.8, 5.0, P > 0.05, paired *t*-test) and 6.6 mmHg (95%CI: 2.5, 10.8, P < 0.05, paired *t*-test) for SBP and DBP respectively.

## 4 Discussion

In this study, we assessed the accuracy of a cuffless continuous BP monitor (SOMNOtouch NIBP) against iBP during CA/PCI and oscillimetric cBP. The accuracy of SOMNOtouch NIBP BtB BP monitor was limited in comparison to simultaneously recorded iBP measured at the level of the brachial artery and cBP. This was noticed for both SBP and DBP measurements. The cBP readings agreed to a large extent with the iBP.

The need for an accurate non-invasive continuous BP monitor has driven research into cuffless BP monitors. One of the concepts underpinning those monitors is PTT [7]. While the use of cuffless BP monitors is not included in the recommended methods of home BP measurements in recent hypertension guidelines [16,17], this field is rapidly evolving with a number of devices already available for self-monitoring of BP [7]. In order to recommend these devices for BP monitoring independent from other cuff-based methods, validation is a necessary prerequisite.

SOMNOtouch NIBP PTT-based BtB BP monitoring has been previously investigated in a number of clinical settings with inconsistent results [[18], [19], [20], [21]]. The device was found to be of acceptable accuracy in a study involving stable participants and following the International Protocol revision 2010 for validation [19]. Similarly, the device delivered acceptable readings in comparison to invasively measured BP in patients admitted to the intensive care unit (ICU) [18] as well as during increasing concentrations of dobutamine infusion [21]. In contrast, in a study conducted in an outpatient clinic comparing SOMNOtouch NIBP with brachial cuff measured BP, the device failed to provide accurate BP readings [20]. The correlation between PTT and estimated BtB BP has been shown to be altered during exercise [22]. Other factors have been shown to impact pulse wave velocity which may affect the feasibility of using PTT based devices in clinical practice [23].

We found a large difference between BP measurements recorded by SOMNOtouch NIBP and recorded by both iBP and cBP measurements. In their recent study, Armstrong et al. showed that invasively measured radial BP and brachial BP were different [24].We showed that at the level of the brachial artery, there were significant differences between the BtB BP calibrated using baseline cBP measurement and both iBP and cBP. As such, our method of comparing different measurement modalities is likely more accurate than studies comparing with radial artery measurements.

This study had some limitations that should be considered when interpreting its results. The measurement of iBP was taken from the contralateral brachial artery. However, the cBP measurement was taken from the ipsilateral side as the BtB BP, and was highly accurate compared to iBP. In addition, most of our patients (71%) had ischemic heart disease which could reflect abnormal vascular elasticity with potential impact on the correlation between PTT and estimated BtB BP [23]. As such, while our findings are directly applicable to similar populations, given the high prevalence of subclinical vascular disease, this may often be the case in those being assessed using continuous BP monitoring.

In conclusion, cuff-less continuous BtB BP monitoring has the potential to provide both patients and clinicians with a tool that could directly influence patient management. However, the accuracy of the technology tested in this study is insufficient for clinical or research use.

## Funding sources

This research was funded by the Dunedin 10.13039/100008235School of Medicine/Basic Medical Sciences Joint Award, University of Otago, New Zealand.

## Author contribution statement

**Mohammed Moharram:** Methodology, Investigation, Formal analysis, Visualisation, Writing - original draft. **Luke Wilson:** Writing - review & editing. **Michael Williams:** Supervision, Investigation, Writing - review & editing. **Sean Coffey:** Conceptualization, Methodology, Supervision, Writing- Reviewing and Editing.

## Declaration of Competing Interest

The authors have no conflicts of interest to declare.
